# Carriage and serotype distribution of *Streptococcus agalactiae* in third trimester pregnancy in southern Ghana

**DOI:** 10.1186/s12884-017-1419-0

**Published:** 2017-07-21

**Authors:** Hans-Christian Slotved, Nicholas T. K. D. Dayie, Josephine A. N. Banini, Niels Frimodt-Møller

**Affiliations:** 1 Department of Bacteria, Parasites and Fungi, Statens Serum Institut, Artillerivej 5, -2300 Copenhagen, DK Denmark; 2 Department of Medical Microbiology, School of Biomedical and Allied Health Sciences, University of Ghana, Accra, Ghana; 3 Department of Clinical Microbiology, University Hospital, Hvidovre, Copenhagen, Denmark

**Keywords:** *Streptococcus agalactiae*, Ghana, Carriage, Pregnancy

## Abstract

**Background:**

The aim of this study was to determine the prevalence of *Streptococcus agalactiae* (group B streptococci, GBS) among healthy, pregnant women attending antenatal care at different study sites in the Greater Accra Region, Ghana.

**Methods:**

Between 2010 and June 2013, recto-vaginal swab samples were collected from pregnant women attending antenatal care from two study sites in southern Ghana. The samples were collected within 35 and 37 weeks of the gestation period. These were inoculated into Todd-Hewitt broth followed by sub-culturing onto a sheep-blood agar plate. Identification was performed on a single subcultured colony. Gram staining was performed, and isolates were evaluated for beta-haemolytic reactions. Furthermore, the isolates were serotyped using the GBS latex serotyping kit.

**Results:**

The carriage rates were found to be 25.5% (95% CI: 19.6–32.1) to 28.0% (95% CI: 21.9–34.8) for the two collection sites. The most common serotypes were serotypes VII and IX. The data showed that women below 20 years of age or above 30 years of age have a significantly (*p* = 0.037) higher risk of carrying GBS compared to women from the age group of 20 to 30 years.

**Conclusions:**

The findings of this study revealed that prevalence of GBS colonization in pregnant women in Greater Accra region is high and comparable to rates observed in South Africa and Western countries. The most prevalent serotypes were serotypes VII and IX, which have not been observed before in West Africa.

## Background

*Streptococcus agalactiae* (group B streptococci, GBS) is a well-known human opportunistic pathogen primarily causing infections in newborns, the elderly and invasive disease in adults with underlying medical conditions [1]. The pathogenesis of the disease in neonates is generally described in two stages: i) Early-onset disease (EOD) which by definition occurs in the neonate during the first six days of life, or ii) late-onset disease (LOD) which is defined as occurring from seven days of life and can develop up to three months of age [1, 2]. Possible clinical manifestations of GBS infection in neoantes are sepis, meningitis and pneumonia [3]. Among adults, GBS is also associated with invasive infections, particularly in elderly persons with underlying medical conditions [1]. The human GBS are currently divided into ten serotypes based on serotype specific antigens and are designated as Ia, Ib, II, III, IV, V, VI, VII, VIII, and IX [1].

Despite advances in medical practice in developed countries, studies have shown that GBS is still considered a life-threatening pathogen; however, antenatal screening of pregnant women between weeks 35 and 37 and subsequent administration of antibiotic prophylaxis to carriers has led to a reduction in GBS diseases in some countries among neonates and women in recent years [4]. In a surveillance study from USA describing the EOD and LOD from 1990 to 2010, the impact of implementation of different GBS disease prevention strategies resulted in a decline in the EOD, although the LOD remains the same [3]. This is, however, not the case in the developing countries and particularly in Africa [1, 4], where GBS is still one of the the leading cause of neonatal diseases. A particular problem with recognizing GBS as an important cause of disease in infants in Africa is the lack of data on the prevalence of colonization in pregnant women in most countries [1]. In recent years, carriage studies from African countries have emerged, showing GBS serotype distribution data from countries such as Gabon, Ethiopia, the Democratic Republic of the Congo, and Zimbabwe [4–7]. In addition, a recent publication from the Central Region of Ghana showed the first carriage rate of GBS among pregnant women [2].

The aim of this study was to determine the prevalence and serotype epidemiology of GBS isolated from pregnant women in southern Ghana.

## Methods

### Study design and study site

This study was carried out in the Greater Accra Region. This region is situated in the southern part of Ghana and has a high population density of 15.8%.

The study was carried out between May 2012 and June 2013 in the Greater Accra region involving two health facilities. Samples were collected from Mamprobi Polyclinic, Accra, and the Dangme West District Hospital, Dodowa, and the laboratory work was performed at the National Public Reference Laboratory, Korle-Bu Teaching Hospital. The Mamprobi Polyclinic is situated in the Ablekuma Sub-metro, which is the largest of the six sub-metros in Accra with a total population of 691,364. The Dangme West District Hospital serves as a major referral center for the people of the Dangme West District, which has a population of 136,622.

### Inclusion criteria

All pregnant women in the last trimester of their pregnancy (35 to 37 weeks of gestation) who consented to participate in the research were included in the studies.

### Collection of samples

Four hundred third trimester pregnant women between 35 and 37 weeks of gestation attending the routine antenatal clinics at the Mamprobi Polyclinic, Accra, and Dangme West District Hospital, Dodowa, were included in the study. A total of 400 vaginal and rectal swab samples were collected between August 2012 and March 2013. Thus, samples from 200 pregnant women were collected from each study site.

### Swab sample collection

Two sterile cotton swab sticks were used to take two different swabs from each person: 1) A swab from the lower vagina and 2) a swab from the rectum. The swabs were collected either by qualified health workers or by the subject themselves after providing them with appropriate instructions [8, 9].

Questionnaires were used to obtain other demographic information. Information retrieved from the women included age, time of pregnancy, parity, previous abortions, level of education, presence of diabetes mellitus, and urinary tract infection (UTI).

### Specimen processing and culturing

An enriched culture medium (ECM) technique was employed. Immediately after the samples were collected under aseptic conditions, the swab sticks were transferred into a selective enrichment broth medium (Todd Hewitt broth) (oxoid LTD) supplemented with gentamicin (8 μg/ml), and nalidixic acid (Sigma Aldrich, 15 μg/ml) and 5% sheep blood to increase the recovery rate of GBS [8, 9]. The collected samples were transported to the laboratory within three hours of sample collection.

In the laboratory, the inoculated broths were incubated overnight (18–24 h) at 37 °C. The broths were subcultured onto 5% sheep blood agar plates (Liofilchem S.R.L. Bacteriology products, Italy) and incubated for 18 to 24 h at 37 °C in 5% CO_2_. Plates with no growth were reincubated for an extra 24 h.

Plates with colonies were examined for their characteristic colonial morphology. Beta-hemolytic and non-hemolytic colonies were also considered. The suspected colonies were Gram stained (Gram positive) and tested for catalase reaction (catalase negative) using 3% hydrogen peroxide. The suspected colonies were further tested using Group B latex agglutination kit (Latex agglutination test, Statens Serum Institut, Copenhagen, Denmark) to confirm the identity of GBS. The confirmed GBS were serotyped using the Strep-B-Latex kit (Statens Serum Institut, Denmark). Strains were cultured for 24 h in Todd Hewitt broth. Ten microlitres from this culture was mixed with specific antisera against serotypes Ia, Ib, and II–IX specific to CPS antigens latex agglutination suspension, and agglutination was read after 5 to 10 s [10].

### Storage

The isolates were stored at −80 °C in a broth containing skimmed milk, tryptone, glucose and glycerol (STGG) [11] at the National Public Reference Laboratory, Korle-Bu.

### Data analysis

All statistical calculations of *p*-values (Either Welch Two Sample t-test or the Chi-square test), odds ratios (OR) and confidence intervals (CI) were performed using R version 3.1.3 for Windows (http://www.r-project.org/).

## Results

### Characteristics of study population

A total of 400 women were enrolled. The characteristics from the two tested sites are presented in Table 1. The two groups showed similar characteristics with regard to mean age, number of abortions, education level and prevalence of urinary tract infections. In addition, no significant differences were found between the two groups except with regards to the number of times a woman had given birth. In Dangme West, the parity was significantly higher (*p* = 0.01) than in Mamprobi. Only two women from Mamprobi Polyclinic had diabetes, and risk assessment was therefore not performed on GBS versus diabetes.

**Table 1:** Grouping of the participants from each site

	Mamprobi (Accra) *N* = 200 (Range)	Dangme West (Dodowa) *N* = 200 (Range)	*P* values or Odds ratio (95% confidence interval)^a^
Maternal age group			
< 20	14^c^	0	
20–30	118	128	
> 30	67	72	
Unknown^b^	1	0	
Mean age	27.99 (15–42)	28.97 (21–44)	*P* = 0.06 (−1.98 – −0.04)
Parity			
0	43	24	
1	56	68	
> 1	78	108	
Unknown^b^	23	0	
Mean parity	1.5 (0–7)	1.9 (0–5)	*P* = 0.01 (−0.62 – −0.08)*
Abortion			
0	114	132	
1	30	48	
> 1	14	20	
Unknown^b^	42	0	
Mean abortion	0.4 (0–6)	0.5 (0–5)	*P* = 0.33 (−0.27–0.09)
Education			
None or Primary	54	56	
Secondary or more	142	144	OR = 1.02 (0.66–1.59), *P* = 0.921
Unknown^b^	4	0	
Urinary tract infection (UTI)			
Yes	11	12	
No	187	120	OR = 0.59 (0.25–1.38) *P* = 0.217
Unknown^b^	2	68	

^a^The *p*-value was either calculated by a chi-square test or a Welch Two Sample t-test. Odds ratios (OR), with confidence intervals of 95% (95% CI), were calculated

^b^Has not been added to the calculation. *The *P*-value is significant

^c^Number of women

### The GBS prevalence for the various sites.

Of the 200 women from Mamprobi (Table 2), 56 of them were carriers of GBS (28.0%, 95% CI: 21.9–34.8), as opposed to 51 women at Dangme West (25.5%, 95% CI: 19.6–32.1). One woman from Dangme West was found to carry multiple GBS serotypes (serotype IV and serotype IX).

**Table 2:** Serotype distribution for each site

GBS serotype	Mamprobi (Accra)^b^ *N* = 200	Dangme West (Dodowa)^b^ *N* = 200
Ia	2	0
Ib	0	0
II	1	0
III	2	4
IV	3	6^a^
V	3	4
VI	0	0
VII	24	20
VIII	3	4
IX	18	14^a^
Total number	56	51 (52 isolates)^a^
Percentage carriage	28.0 95% CI (21.9–34.8)	25.5.0 95% CI (19.6–32.1)

^a^One person from Dangme West was positive for two different GBS serotypes, serotype IV and serotype IX

^b^Number of isolates

Of the 56 positive samples from Mamprobi, 44 women had positive GBS vaginal samples, four had both positive vaginal and rectal samples and eight had rectal positive samples only. In Dangme West, 40 women had positive vaginal samples only, two had both vaginal and rectal positive samples, and nine had positive rectal samples only.

Table 2 shows the serotype distribution for both sites. Eight of the ten known GBS serotypes were found in the study, although not equally represented at all four sites. Serotype Ib and VI was not found at the sites. The study showed a serotype distribution in which serotypes VII and IX were the dominant serotypes found at both study sites. At Mamprobi, 42.9% of the isolates were serotype VII, while 32.1% were serotype IX. In Dangme West, 38.5% of the isolates were serotype VII, while 26.9% were serotype IX.

### Association between social demographic factors and GBS colonization

#### Risk factors for Mamprobi

Age was found to be a risk factor (Table 3). Women below 20 years of age or above 30 years of age have a significantly (*p* = 0.037) higher risk of carrying GBS compared to women from the age group of 20 to 30 years. Individual risk factors such as parity, abortions, the level of education, and urinary tract infections did not show to have any effect on the risk of carrying GBS.

**Table 3:** Risk factors for Mamprobi (Accra)

	GBS carriage (*N* = 56)	No GBS carriage (*N* = 144)	OR	95% CI	*P* values^a^
Maternal age group					
< 20 and >30	29	51	1		
20–30	27	92	0.52	0.28–0.97	*P* = 0.037*
Unknown^b^	0	1			
Parity					
0	11	32	1		
> 0	38	96	1.15	0.53–2.52	*P* = 0.723
Unknown^b^	7	16			
Abortion					
0	42	72	1		
> 0	14	30	0.80	0.38–1.68	*P* = 0.554
Unknown^b^	0	42			
Educational level					
None or primary	15	39	1		
Junior high school, Senior high school and Post secondary	40	102	1.02	0.51–2.05	*P* = 0.957
Unknown^b^	1	3			
Urinary tract infections (UTI)					
Yes	3	8	1		
No	52	135	0.97	0.25–3.81	*P* = 0.969
Unknown^b^	1	1			

^a^The *p*-value was calculated by a chi-square test. Odds ratios (OR) with confidence intervals of 95% (95% CI) were calculated

^b^Has not been added to the calculation. *The *P*-value is significant

#### Risk factors for Dangme west

Both age (*p* < 0.001) and parity (*p* = 0.006) (Table 4) were found to be significant risk factors for GBS carriage, while abortion, the level of education, and urinary tract infections were not found to be significant risk factors.

**Table 4:** Risk factors for Dangme West (Dodowa)

	GBS carriage (*N* = 51)	No GBS carriage (*N* = 149)	OR	95% CI	*P*-value^a^
Maternal age group					
< 20 and >30	31	41	1		
20–30	20	108	0.24	0.13–0.48	*P* < 0.001*
Unknown^b^	0	0			
Parity					
0	0	24	1		
> 0	51	125	10.31^c^	1.36–78.14	*P* = 0.006*
Unknown^b^	0	0			
Abortion					
0	28	104	1		
> 0	23	45	1.90	0.99–3.65	*P* = 0.053
Unknown^b^	0	0			
Education level					
None or primary	12	44	1		
Junior high school, Senior high school and Post secondary.	39	105	1.36	0.65–2.84	*P* = 0.41
Urinary tract infections (UTI)					
Yes	3	12	1		
No	28	120	1.10	0.28–4.32	*P* = 0.897
Unknown^b^	20	17			

^a^The *p*-value is calculated by a chi-square test. Odds ratios (OR), with confidence intervals of 95% (95% CI), were calculated

^b^Has not been added to the calculation. *The *P*-value is significant

^c^Where zeros cause problems with computation of the odds ratio, 1 was added to all four cells

## Discussion

The prevalence of GBS among pregnant women has been studied in many countries; however, very little is known about GBS carriage among pregnant women in developing countries [12]. In recent years, carriage studies to determine the prevalence and serotype epidemiology among pregnant women have emerged in developing countries. The review study by Dagnew et al. [12] shows an overview of some of the incidence studies performed in developing countries. In the review, the GBS prevalence ranged from 12% in Asia/Pakistan to 22% in the Middle East/North Africa. Our study provides GBS prevalence data and serotype distribution for southern Ghana, adding further data to the GBS prevalence in Ghana. A previous study from Ghana presented the prevalence of GBS in the Ashanti region [2] where they found an overall carriage rate of 19.1%, with 23.1% carriage in Kumasi and 18% in Pramso. These carriage rates are lower than what is reported at Mamprobi and Dangme West in this study (28.0% and 25.5%, respectively) (Table 2). However, both studies showed that the prevalence of GBS is generally high in Ghana. A similar high GBS prevalence has also been observed in studies from other African countries. In a study from Gabon, the GBS carriage rate was reported to be 19% [13]. Mitima et al. [6] found a GBS prevalence of 20% in the Congo. Another study showed a carriage rate of 20.2% in Kenya and 23.1% in South Africa [14]. In general, all these GBS prevalence studies documented a carriage rate around 20% or above; however, there are also studies reporting very low prevalences as seen in the study by Woldu et al. [7], who found a GBS prevalence of 7.2% in Ethiopia, and de Steenwinkel et al. [15] who reported a GBS prevalence of 1.8% among pregnant women in Maputo, Mozambique. The reason for the differences in the GBS prevalence between the studies can be attributed to differences in the methodology as suggested by Dagnew et al. [12] In the study by Cools et al. [14], only vaginal swab samples were collected, which they suggest might be responsible for the lower carriage rate, compared to isolation of GBS from both vaginal and rectal swabs. If all the positive rectal swab samples were removed in this study, the carriage rate would have been 24% for Momprobi and 21% for Dangme West. This would indicate a reduction in the carriage rate by approximately 4%. It has however also been shown that specimens collected by swabbing the lower vagina and/or the rectum yield adequate samples for screening [8]. In the study from the Ashanti region in Ghana, they only tested and found five different serotypes, of which serotypes Ia, V, and III were the most common serotypes, particularly in the rural areas [2]. The carriage data that we have collected (Table 2) can be grouped into urban area site (Mamprobi), while Dangme West can be considered a rural site. The findings in this study with respect to GBS serotype distribution are similar to that of Vinnemeier et al. [2] in Kumasi. We furthermore found a similar carriage rate, when comparing the prevalence between rural site (Dangme West, 25.5% carriage) and urban site (Mamprobi, 28.0%). It could have been interesting to see whether the five non-typable serotypes from the Vinnemeier et al. [2] study could have been identified as belonging to either serotype VII or IX, if the isolates had been tested for all 10 GBS serotypes. The GBS serotype distribution found at the Mamprobi and Dangme West sites, where serotypes VII and IX were the dominating serotypes at both sites (Table 2), are very different from what has been observed in previous GBS studies in Africa. Several studies from Africa have found that the predominant GBS serotypes were found among the serotypes Ia, Ib, II, III, and serotype V, while serotypes IV, VI, VII, VIII, and serotype IX were not seen [2, 4, 16, 17]. In a recent study, however, Cools et al. [14] found a relatively high prevalence of serotypes VI, VII, and VIII in Kenya and serotypes IV, VI and VIII in South Africa, while they did not detect serotype Ib. The reason for these differences in the GBS serotype distribution between the studies from African countries, may be attributed to the geographical location, the typing procedures or other variables [18]. However, testing for all ten known serotypes is important, since serotypes normally thought to be prevalent only in certain parts of the world, such as serotype VIII, might also be found at other sites in other parts of the world as seen in the study by Ekelund et al. [19]. In this study, we did not observe any non-typable serotypes (Table 2). It is not uncommon to see studies with no NT isolates [20]. Often NT isolates are found in studies, which do not test for all 10 known serotypes, such as Vinnemeier et al. [20]. We can not explain why we did not find any NT isolates in this study using only serological methods, however because we tested for all ten serotypes, we did reduce the possibilities of NT isolates.

At present, there are two vaccines in phase II trials [14, 18], which will cover the serotype Ia, Ib and III. However, as seen in both our studies and other studies [2, 4, 14], these GBS vaccines would not protect a large number of women in several African countries.

In this study the maternal age was found to be a significant risk factor for both sites, whereas parity was found to be a significant risk factor for Dangme West (Tables 3 and 4). The number of abortions, the level of education, and urinary tract infections were not found to be significant risk factors at any of the study sites. Risk factors for GBS carriage do vary, depending on the study. In a study from DR Congo [6], it was found that age and parity were not risk factors, while education, UTI and abortion were found to be risk factors. In another study from Gabon [13] only illiteracy was found to be a significant risk factor. In the study by Cools et al. [14], they found a statistically significant age-group dependent GBS association in the non-pregnant women, with the lowest and the highest GBS carrier rates in the youngest and the oldest age groups. However, they also found that this age dependent risk could not be seen in pregnant women. According to some studies incidence of invasive disease are increasing in adults with underlying medical conditions such as diabetes mellitus [16]. Diabetes mellitus was therefore investigated as a risk factor, however our data was limited to perform any calculations. Larger GBS carriage studies are needed to give a clearer picture of which risk factors are important in African countries.

## Conclusion

In conclusion, this study found a high prevalence of GBS in pregnant women in Southern Ghana, similar to the prevalence found in other parts of the country. The GBS prevalence is furthermore very similar to other countries in the region and elsewhere in the United States and other Western countries. Regarding serotype distribution, we identified a different serotype distribution pattern in this study compared to what was observed in the study by Vinnemeier et al. [2]. As also observed in the study by Cools et al. [14], the current phase II GBS vaccines focusing only on serotypes Ia, Ib, and III would not protect a large number of the women participating in this study.

## Abbreviations

CI: Confidence interval
ECM: Enriched culture medium
EOD: Early-onset disease
group B Streptococci, GBS: *Streptococcus agalactiae*
LOD: Late-onset disease
OR: Odds ratio
UTI: Urinary tract infections
